# 

**Published:** 1876-11

**Authors:** 


CONTENTS.

| | PAGE |
|---|---|
| PERIODICAL LITERATURE | 455 |
| PUBLIC SCHOOLS | 461 |
| HAMMER ON | 463 |
| PHYSICAL TRAINING | 465 |
| LIVING ON EXCITEMENT | 473 |
| THE HOURS MOST FATAL TO LIFE | 474 |
| THE BATTLE AND THE BURTHENS OF LIFE | 475 |
| POISONOUS VISITING CARDS | 476 |
| THE HARDEST MODE TO DIE | 477 |
| GOING TO THE SOUTH | 478 |
| SIR ASTLEY COOPER'S BATH | 480 |
| KEEPING THE TEETH CLEAN | 482 |
| THE LUNGS | 483 |
| BRANDY AND THROAT DISEASE | 484 |
| HEALTH OF CITIES | 485 |
| INCONSIDERATIONS | 486 |
| MY HOME IN NEW ENGLAND | 487 |
| OLDBOY BROTHER'S NEAREST RELATIVE | 487 |

